# Modern contraception methods usage trend among Egyptian females and its determinant factors: results from five national surveys

**DOI:** 10.1186/s12889-026-28100-x

**Published:** 2026-06-17

**Authors:** Samar Fares, Manal Naguib, Abeer Attia, Saeed Soliman

**Affiliations:** 1 Family Medicine Department, Kasr Alainy Faculty of Medicine, Cairo University, Cairo, Egypt; 2 Clinical pharmacist, Ministry of Health and Population, Alexanderia, Egypt; 3 Department of Public Health and Community Medicine, Kasr Alainy Faculty of Medicine, Cairo University, Cairo, Egypt; 4 Kasralainy Faculty of Medicine, Cairo University, Cairo, Egypt

**Keywords:** Contraception, Birth Control, Egypt, Females, Trends, Determinants, DHS.

## Abstract

**Background:**

Family planning is considered one of the most effective health-promoting strategies, with the ability to prevent approximately 30% of maternal mortality and 10% of child mortality. In Egypt, there have been remarkable governmental efforts and legislation for over 50 years. This study aims to conduct a secondary data analysis of existing survey datasets to compare and explore trends in the prevalence of modern contraception use across five national surveys conducted between 2000 and 2014 in Egypt.

**Methods:**

This secondary data analysis relies on the five most recent Egyptian Demographic and Health Surveys (EDHS) conducted in the years 2000, 2003, 2005, 2008, and 2014. EDHS is a nationally representative survey following a multistage stratified cluster sampling design, producing estimates generalisable to the Egyptian national population and providing information on important maternal, child health and family planning indicators. Ever-married females aged 15–49 years were included, and those who were pregnant, infecund, or never-married were excluded. The total population included in the five surveys was 82,458, while the eligible females for this analysis were 51,329, for whom modern contraceptive prevalence use and factors associated with it, including sociodemographic, birth-related, and access to health care factors, were assessed.

**Results:**

The mean age was 32.8 (± 8.1) years. The mean modern contraception (MCC) usage was 74.4% over all surveys. The most recent survey (EDHS 2014) showed a decline to 73.1%, down from 75.4% in 2008. Women aged 35 to 44 had a higher chance of having MCC (OR 95% CI 1.42 (1.18–1.70)), while women aged 15 to 24 had a lower chance (0.73 (0.60–0.88)). Women with middle and high household wealth indices, professional working women, and women with more than three living children showed higher odds of using MCC compared to their counterparts, with odds ratios of 1.33 (1.15–1.55), 1.88 (1.53–2.31), 1.25 (1.04–1.51), and 1.23 (1.07–1.42). Mode and place of last delivery, together with the source of contraception method, play a vital role in MCC use [1.23 (1.07–1.42), 1.31 (1.03–1.68) & 2.49 (2.19–2.83)].

**Conclusion:**

Although MCC usage averaged 74.4% across the five surveys, this study highlights persistent differences in modern contraceptive use in Egypt, with notable gaps by age, residence, and socioeconomic status.

## Introduction

Family planning is deemed to be one of the most effective health-promoting strategies, with the ability to prevent around 30% of maternal mortality and 10% of child mortality [1]. Egypt has struggled with rapid population growth for many years. By 2017, the total number of Egyptians, both inside and migrating outside the country, reached about 104 million [2]. The contraceptive prevalence among married or in-union women aged 15–49 years improved worldwide from 55% in 1990 to 64% in 2012 [3]. The Eastern Mediterranean Region (EMR) lies only second after Africa, with the least prevalence of contraceptive use (48%) and the highest unmet needs for family planning worldwide [4]. Several surveys in rural Egyptian areas and in Alexandria suggested that 12.6% and 16.28% of currently married women aged 15–49 still have an unmet need for contraception [5, 6]. This problem is particularly marked in the first year after childbirth, and about 40% of women say they intend to use contraception but have not yet started. Notably, research from rural Egypt in 2008 found that approximately 29% of pregnancies were unintended, a proportion considerably lower than the global average of over 40%, suggesting a relative strength of Egypt’s family planning programme in this context, though unmet need remains a concern [7]. Several studies have suggested that both organisational health-system constraints and social barriers contribute to unmet needs for contraceptives, and much of this evidence is based on health-care providers’ perspectives on barriers towards contraceptive use [7, 8].

In Egypt, the overall contraceptive prevalence rate (including both modern and traditional methods) is 60% among married women of reproductive age, as reported by the WHO [4]. In Egypt, there had been remarkable governmental efforts and legislation for over 50 years, even non-governmental efforts started before then by the Coptic Evangelical Organization for Social Service (CEOSS), established in 1954, as it adopted health promotion programs including family planning [9].

Since the 1970s, a government-headed national family planning programme has been funded and assisted by several parties, achieving significant results in increasing family planning service coverage and contraceptive use while decreasing total fertility rates and unmet needs for family planning [5]. However, Egypt’s family planning programme had been vulnerable to political problems, as in the public sector, modern contraceptive methods, including pills, IUDs, injectables, and condoms, are provided free of charge or at subsidised cost through Ministry of Health and Population (MOHP) facilities and Egyptian Family Planning Association (EFPA) clinics without requiring a prescription. Access policies were broadly consistent throughout the 2000–2014 study period, though programme continuity was temporarily disrupted following the January 25th, 2011 revolution’s change, when the programme was still recovering. Egypt is also committed to the goals of Family Planning 2030 (FP2030), a global partnership to advance women’s rights and access to voluntary family planning [6].

In Egypt, modern contraceptive services are provided via a dual public-private system. offers subsidised counting for over half of modern contraceptive provision according to the 2014 EDHS [10]. The private sector, on the other hand, is becoming more important, especially for urban and wealthier women [10, 11]. The EDHS 2014 data show that the source of contraception is a major factor in the use of modern methods, with big differences in how many people use them in the public and private sectors [10, 11]. Access differs based on location and income level, with known differences between women in cities and those in rural areas and between women in different wealth quintiles [11].However, whether these differences reflect supply-side access constraints or demand-side factors cannot be determined from survey data alone [11]. Comprehending this service delivery landscape offers essential context for analyzing the trends and determinants explored in this study.

In Egypt, family planning service utilization and geographic availability of contraceptive methods are monitored periodically using various tools, including the Egypt Demographic and Health Survey (EDHS) [12]. All reports of EDHS are available, but a cross-survey comparison of contraception usage has not been done before. These five surveys represent the only publicly available nationally representative microdata on contraceptive use in Egypt for this period. Unlike many regional and international studies that use all reproductive-aged women as the denominator, this analysis is restricted to currently married, non-pregnant, non-infecund women, the population presumed to be at risk of pregnancy and therefore the most policy-relevant denominator for assessing unmet contraceptive need. Thus, this study aims to perform secondary data analysis of existing surveys to compare and explore trends in modern contraception prevalence usage across five national surveys conducted in Egypt between 2000 and 2014. Also, from the latest survey, we will examine the determinants of modern contraception usage. It performs a compiled analysis that leverages the large sample sizes of multiple existing surveys to illustrate trends in modern contraceptive use among Egyptian women across the 2000–2014 period, helping stakeholders outline future plans based on these trends.

## Methods

### Data sources

This secondary data analysis relies on data from the most recent five Demographic and Health Surveys (EDHS) conducted in the years 2000, 2003, 2005, 2008, and 2014 [11, 13–16]. The Egypt Demographic and Health Surveys (EDHS) are conducted at irregular intervals, depending on national priorities and funding availability. The most recent publicly available EDHS dataset at the time of this analysis is the 2014 survey. The EDHS provides information on the most important health and demographic indicators, including maternal and child health and family planning. The wealth index was developed using a systematic approach involving principal components analysis (PCA) applied to household characteristics and asset data collected across multiple demographic health surveys. Initially, a common set of indicators applicable to both urban and rural households was identified, with categorical variables transformed into dichotomous indicators. PCA was employed to generate wealth scores for households, which were then refined into separate factor scores for urban and rural areas using area-specific indicators. The final wealth index was created by adjusting these scores through regression on the common factor scores, resulting in a standardized index with a mean of zero and a standard deviation of one, allowing for the classification of households into five wealth quintiles. Households were then ranked according to their total asset scores, leading to the division of the population into quintiles. This asset-based wealth index has been validated against other socio-economic indicators, demonstrating its reliability in reflecting long-term living standards and correlating well with consumption expenditure data from various countries. Data use was approved by Inner City Fund International (ICF), and Institutional Review Board (IRB) permission was granted by the DHS program.

### Sampling

EDHS is a country representative that follows a multistage stratified cluster sampling complex sample design. In general, each survey uses the most recent census to divide the country into strata, and within the strata, some primary sampling units (PSUs) are selected. Finally, many households are selected and surveyed from each PSU. Details of the EDHS sampling procedure are described in each survey [17]. Using the Integrated Public Use Microdata Series–demographic health survey (IPUMS-DHS) merging tool, we were able to combine the 5 surveys’ data sets [17].

### Sample size

The 2000 and 2005 surveys systematically selected about 22,807 households from 682 primary sampling units, yielding interviews with nearly 19,565 ever-married women aged 15–49. The 2003 interim survey followed a similar design but with a smaller sample size, focusing on fertility and family planning indicators to provide updated estimates between major survey rounds. In 2008, the design emphasized precision at the domain level, recommending a minimum of 1,000 cases per domain, achieved through 25–30 households per primary sampling unit and at least 40 PSUs per domain. The 2014 survey expanded to a four-stage design, selecting 884 PSUs (after excluding North and South Sinai for security reasons), subdividing them into parts and segments, and systematically sampling about 29,172 households, resulting in 24,158 completed women’s interviews.

### Data collection in EDHS surveys

Data collection for the demographic health surveys involved a comprehensive and structured process to ensure the accuracy and reliability of the information gathered. Initially, pretests were conducted in select households to refine the questionnaires based on feedback from field staff. Recruitment of interviewers and field editors focused on recent university graduates, minimizing bias by selecting candidates without prior anemia testing.

Training programs for field staff included lectures on interview techniques, survey content, and hands-on practice, supplemented by assessments to ensure competency. Fieldwork was organized into teams comprising supervisors, field editors, interviewers, and staff for anthropometric measurements assigned to specific geographic areas. Quality control measures were implemented, including reinterviews and callbacks, to verify data accuracy and identify errors. Throughout the process, data collection utilized well-structured questionnaires, capturing a range of demographic, health, and wealth-related information and maternal and child health data [11, 13–16].

### Eligibility criteria

**Fig. 1:**
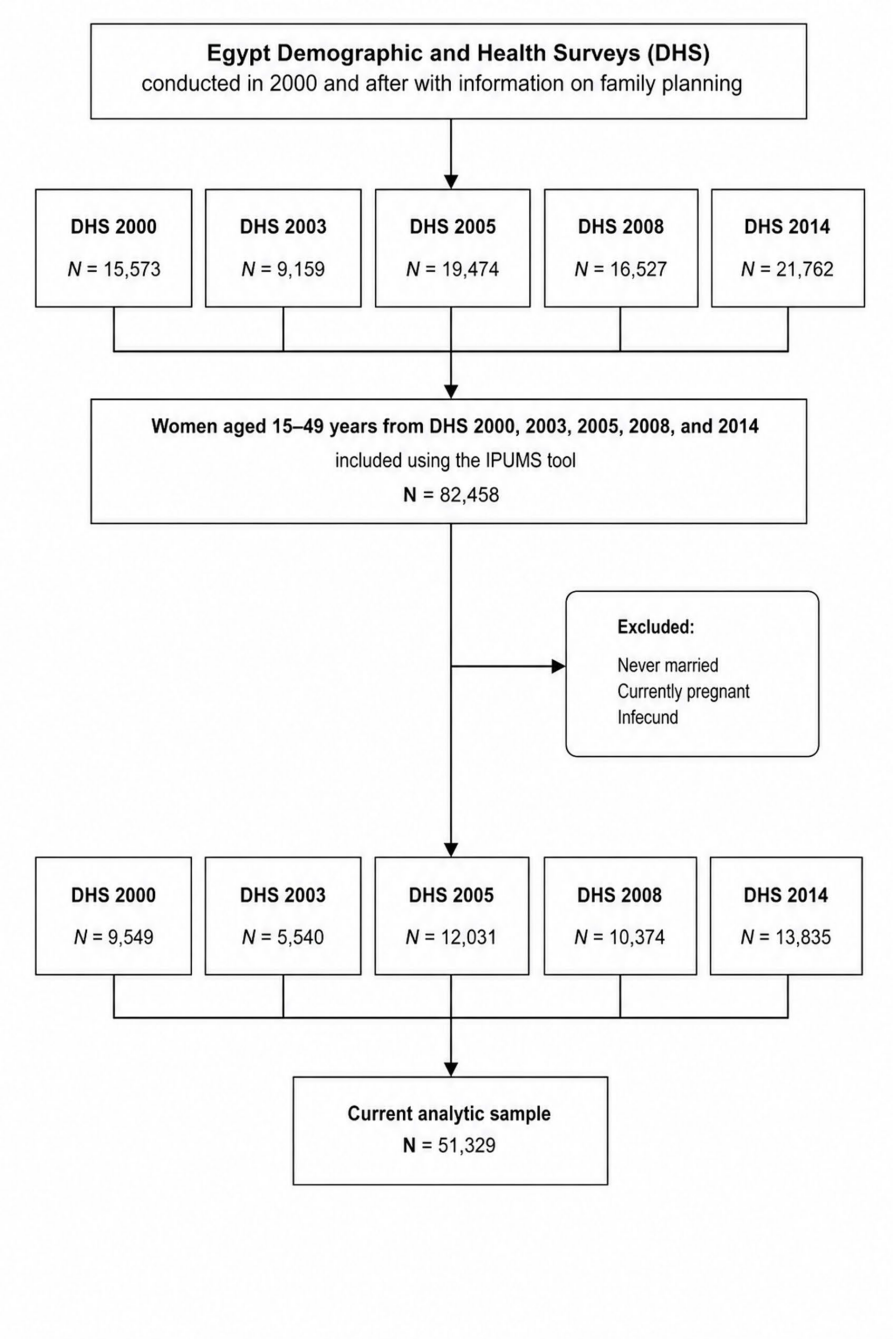
Detailed description of the study flow and sample selection

Ever-married females, after the inclusion of currently married women aged 15 to 49 years, were included. We excluded women who had been pregnant at the time of the survey, infecund females, and never-married females. It is acknowledged that intrinsic fecundity also varies across age groups, with women approaching the menopausal transition (age 45–49) having naturally reduced fertility; however, the DHS infecundity proxy captures this physiological variation as part of the infecund exclusion criterion. The DHS infecundity proxy classifies women as infecund if they meet one or more of the following criteria: [1] self-report of being unable to conceive due to medical condition [2], absence of menstrual periods (amenorrhea) combined with not being postpartum or currently pregnant, or [3] no live birth and no contraceptive use in the five years preceding the survey, in the absence of reported intention to conceive. This classification is applied consistently across all five EDHS waves using the DHS standard variable ‘infecund.’ In alignment with EDHS analytical conventions and the sociocultural context of Egypt and the Eastern Mediterranean Region, where sexual activity is culturally confined to marriage, and premarital sexual activity is both uncommon and underreported, our analysis was restricted to married women *aged 15–49 years*. Consequently, married women represent the population exposed to the risk of pregnancy and eligible for contraceptive use. This approach inherently excludes women who are presumed to have not initiated sexual activity prior to marriage, consistent with the sociocultural context of Egypt and EDHS analytical conventions and including never-married women. The total number of eligible females for this analysis was 51,329 women; a detailed description of the study flow and sample selection is illustrated in Fig. 1.

### Variable selection

#### Outcome variable

The primary outcome of interest was the use of modern contraceptives. The dependent variable was therefore a binary indicator of current use (within one month) of any modern contraceptive method (Yes/No) among married women of reproductive age (15–49 years). The prevalence of modern contraceptive use was calculated as the proportion of women whose response to this variable was ‘Yes.’ The contraception prevalence was calculated for every survey independently and then collectively across the five surveys using the following formula:

Number of women 15–49 using a modern contraceptive method / total number of women 15–49 × 100 [18].

The EDHS survey defines a modern contraceptive method as any of the following: female or male sterilization, pill, intrauterine device, injection, implants, diaphragm, foam or jelly, male or female condom, lactational amenorrhea (LAM), or emergency contraception [18]. Women currently using any of the modern methods were coded “yes” while women not using contraceptives or using traditional methods, comprising periodic abstinence and withdrawal (methods with limited or no established clinical efficacy under typical use for the calendar- and symptom-based approaches available during the study period), and folk methods (locally described or spiritual methods of unproven effectiveness, such as herbs and amulets), as classified by the DHS Program [18], were coded ‘no’.

Note: Digital fertility awareness methods with published typical-use failure rates (e.g., Natural Cycles, FDA-cleared in 2018) were not available during the 2000–2014 study period and were therefore not relevant to this dataset.

Regarding the controversy about LAM as an MCC method, the World Health Organization (WHO) and the DHS Program classify LAM as an effective modern contraceptive method when the three LAM criteria are met: The woman is amenorrhoeic; she is exclusively or nearly exclusively breastfeeding, and her infant is less than 6 months old [19]. In the DHS instrument, women self-report their current contraceptive method; they are not administered a clinical checklist of LAM criteria at the time of interview. Women who reported ‘breastfeeding/LAM’ as their current method were classified as modern contraceptive users, consistent with DHS standard classification [18]. This may include some women who do not meet all three WHO LAM criteria, a potential source of misclassification acknowledged as a limitation of survey-based LAM reporting.

#### Independent variables

Based on the literature [10, 20–23], the demographic variables include the husband’s and the woman’s age, residence (urban or rural), job, educational level (primary, secondary, or higher), and wealth categories. Marriage variables include age at first marriage and birth-related variables, including premarital medical examination, antenatal care (percent of women receiving obstetric care distributed according to the type and skill of the provider), mode (if delivery was by cesarean section), place of last birth (governmental sector, private sector, or home), total children alive, and health check after last birth (women with a live birth in the last 2 years who had a health check after delivery).

To ensure meaningful interpretation of demographic patterns related to contraceptive use, some independent variables were recategorized. All age-related variables were recategorized into life-stage–appropriate groups. Age was categorized into four groups (15–24, 25–34, 35–44, and 45–59 years), reflecting commonly used reproductive life stages that correspond to differences in fertility intentions and contraceptive behavior.

Age at first marriage was also recoded into four categories (8–14, 15–19, 20–30, and 30–49 years) to distinguish child and early marriages from typical and late marriage patterns, a distinction that is particularly relevant in studies of reproductive health and contraceptive uptake.

The ***household wealth index*** variable was initially provided in five quintiles (poorest, poorer, middle, richer, richest). For this analysis, we recoded the quintiles into **three wealth terciles** to obtain more balanced groups and to facilitate interpretation. Specifically, we **merged the two lowest quintiles** (Poorest and Poorer) to form the *Poor* category, retained the middle quintile as Middle, and **combined the two highest quintiles** (Richer and Richest) to form the Rich category. These terciles were used consistently in the descriptive and regression analyses.

### Statistical analysis

#### Data was analyzed using Statistics and data, a statistical software for data science

(STATA)^®^ version 15 [24]. In all analyses, complex sample designs and sample weights were applied using the the survey prefix command in STATA software (SVY) set command as advised by the DHS website [17]. Specifically, the individual women’s weight variable (v005, divided by 1,000,000) was applied to all prevalence estimates and regression models to account for unequal selection probabilities and non-response, ensuring results are representative of the target population. Descriptive statistics were used to describe the frequencies and weighted percentages of categorical variables and the mean (SD) for continuous variables. The Rao-Scott chi-square test describes the association between categorical variables, and the Student t-test explores the mean difference of continuous variables.

Univariate logistic regression analysis was used to find statistically significant factors for contraception use. Significant variables were then entered into the multivariate logistic regression model, and adjusted and unadjusted odds ratios were reported. For that model, the reference category for each variable was set to the most clinically or programmatically meaningful group, specifically, the group with the lowest anticipated exposure to the outcome, consistent with standard DHS analytical practice and the WHO guide to DHS statistics. In all analyses, the significance level was set at < 0.05.

Despite the expanded sampling design of the 2014 EDHS, which introduced a four-stage approach and excluded North and South Sinai for security reasons, inclusion of the 2014 wave in trend analysis is justified by three considerations: [1] the DHS Program applies a consistent set of standardized questionnaire modules, variable definitions, and quality assurance protocols across all EDHS waves, enabling cross-survey comparability of the contraceptive use outcome variable; [2] DHS survey weights (v005/1,000,000) adjust for differential selection probabilities within each survey, partially correcting for design differences; and [3] excluding 2014 would eliminate the only post-2011 Revolution data point, which is of primary policy relevance. Nonetheless, readers should interpret the 2014 estimates with awareness that methodological differences may contribute to observed changes alongside true population-level shifts.

## Results

A total of 51,329 Egyptian women who met the eligibility criteria from all surveys were included in this analysis. Full descriptive statistics are presented in Table 1. The age range was 15 to 59 years, and the mean (± SD) was 32.8 (± 8.1) years. A greater proportion of participants were in the 25-44-year age group (73.3%, *n* = 37604), living in rural areas (58.1%, *n* = 28428). Just under half of the participants were residing in households classified as rich by wealth index (44.4%, *n* = 22830), while 34.5% (*n* = 18425) were classified as poor. Educational attainment showed that 44.0% (*n* = 22794) had secondary-level education, while 11.9% (*n* = 6032) had higher (university degree level) education. Most participants had no work (80.7%, *n* = 41,347) or a professional category job (12.3%, *n* = 6,492), while 7% (*n* = 3,467) were employed in manual or skilled trade occupations (e.g., agricultural, craft, or production workers, as classified by the DHS occupational categories). 62.9% (*n* = 32335) were married before the age of 20 years.

**Table 1:** Modern Contraception Use and Sociodemographic Characteristics of Married Women in the pooled 5 DHS data (*n* = 51329) and in DHS 2014 survey in Egypt (*n* = 13835)

	Modern Contraception Use					
	Pooled data (5 DHS surveys)			DHS 2014 survey		
Sociodemographic Characteristics	Yes *n* (%)	No *n* (%)	*p*-value	Yes *n* (%)	No *n* (%)	*p*-value
Residency						
Urban	17,578 (76.8%)	5323 (23.2%)	< 0.001	4769 (75.2%)	1570 (24.8%)	< 0.001
Rural	19,816 (69.7%)	8612 (30.3%)		5190 (69.2%)	2306 (30.8%)	
Age at first marriage						
8–14	2975 (75.0%)	990 (25.0%)	< 0.001	504 (73.5%)	182 (26.5%)	< 0.001
15–19	16,914 (72.9%)	6288 (27.1%)		4212 (73.2%)	1540 (26.8%)	
20–30	17,175 (73.3%)	6260 (26.7%)		5137 (71.8%)	2016 (28.2%)	
30–49	330 (45.4%)	397 (54.6%)		106 (43.4%)	138 (56.6%)	
Age group of respondents						
15–24	4627 (52.1%)	4246 (47.9%)	< 0.001	1088 (51.9%)	1010 (48.1%)	< 0.001
25–34	14,905 (73.5%)	5378 (26.5%)		4192 (70.5%)	1751 (29.5%)	
35–44	14,104 (81.4%)	3217 (18.6%)		3608 (81.3%)	831 (18.7%)	
45–59	3758 (77.5%)	1094 (22.5%)		1071 (79.0%)	284 (21.0%)	
Current husband age						
15–24	9256 (63.1%)	5409 (36.9%)	< 0.001	2770 (62.7%)	1648 (37.3%)	< 0.001
25–34	566 (38.1%)	919 (61.9%)		155 (44.5%)	193 (55.5%)	
35–44	14,682 (77.8%)	4198 (22.2%)		3568 (75.9%)	1134 (24.1%)	
45–59	12,890 (79.1%)	3409 (20.9%)		3466 (79.4%)	901 (20.6%)	
Household wealth index						
Poor	12,545 (68.1%)	5880 (31.9%)	< 0.001	3175 (67.7%)	1515 (32.3%)	< 0.001
Middle	7350 (73.0%)	2724 (27.0%)		1879 (71.2%)	760 (28.8%)	
Rich	17,499 (76.6%)	5331 (23.4%)		4905 (75.4%)	1601 (24.6%)	
Summary of educational achievement						
No education	11,007 (71.0%)	4495 (29.0%)	< 0.001	2003 (71.7%)	791 (28.3%)	0.85
Primary	5254 (75.0%)	1747 (25.0%)		960 (72.9%)	356 (27.1%)	
Secondary	16,694 (73.2%)	6100 (26.8%)		5446 (71.9%)	2130 (28.1%)	
Higher	4439 (73.6%)	1593 (26.4%)		1550 (72.1%)	599 (27.9%)	
Husband’s education						
No education	7102 (71.2%)	2879 (28.8%)	< 0.001	1423 (73.0%)	527 (27.0%)	0.004
Primary	7004 (75.2%)	2305 (24.8%)		1402 (74.6%)	478 (25.4%)	
Secondary	17,099 (71.8%)	6718 (28.2%)		5358 (70.8%)	2210 (29.2%)	
Higher	6172 (75.3%)	2028 (24.7%)		1775 (72.9%)	661 (27.1%)	
Last birth to interview (years)						
< 1 year	5042 (72.9%)	1872 (27.1%)	< 0.001	1449 (73.7%)	517 (26.3%)	< 0.001
1–3 years	10,471 (73.4%)	3790 (26.6%)		2784 (71.4%)	1115 (28.6%)	
> 3 years	21,868 (80.8%)	5195 (19.2%)		5723 (79.7%)	1458 (20.3%)	
Number of living children						
None	21 (0.7%)	3198 (99.3%)	< 0.001	4 (0.5%)	814 (99.5%)	< 0.001
1 to 3 children	23,864 (76.1%)	7487 (23.9%)		6963 (74.8%)	2350 (25.2%)	
> 3 children	13,509 (80.6%)	3250 (19.4%)		2992 (80.8%)	712 (19.2%)	
Premarital medical exam						
No	22,765 (74.6%)	7749 (25.4%)	< 0.001	7470 (75.9%)	2367 (24.1%)	< 0.001
Yes	1465 (55.6%)	1169 (44.4%)		1205 (54.3%)	1014 (45.7%)	
Source for family planning						
Government	20,795 (78.6%)	5673 (21.4%)	< 0.001	5253 (69.1%)	2349 (30.9%)	< 0.001
Private	16,242 (90.2%)	1760 (9.8%)		4660 (85.6%)	783 (14.4%)	
Other source	326 (55.6%)	260 (44.4%)		27 (31.8%)	58 (68.2%)	
Caesarean section (last birth)						
No	15,111 (73.7%)	5387 (26.3%)	0.002	2605 (72.6%)	981 (27.4%)	0.23
Yes	6156 (72.5%)	2337 (27.5%)		3071 (71.0%)	1252 (29.0%)	
Place of delivery (last birth)						
Home	6128 (70.1%)	2618 (29.9%)	< 0.001	605 (69.5%)	266 (30.5%)	0.068
Governmental	5577 (75.6%)	1802 (24.4%)		1512 (73.3%)	551 (26.7%)	
Private sector	9441 (74.2%)	3276 (25.8%)		3515 (71.4%)	1407 (28.6%)	
Others	133 (79.2%)	35 (20.8%)		44 (83.0%)	9 (17.0%)	
Received prenatal care						
No	5323 (69.9%)	2292 (30.1%)	< 0.001	492 (67.2%)	240 (32.8%)	0.013
Yes	15,951 (74.6%)	5438 (25.4%)		5184 (72.2%)	1993 (27.8%)	
Professional health check after birth						
No	8686 (72.3%)	3336 (27.7%)	0.092	871 (68.4%)	403 (31.6%)	0.01
Yes	7449 (73.5%)	2692 (26.5%)		4283 (70.1%)	1830 (29.9%)	
Woman current work category						
Professional	5127 (79.0%)	1365 (21.0%)	< 0.001	1237 (77.7%)	356 (22.3%)	< 0.001
Manual	2634 (76.0%)	833 (24.0%)		530 (76.1%)	166 (23.9%)	
No work	29,616 (71.6%)	11,731 (28.4%)		8192 (70.9%)	3354 (29.1%)	
Husband current work category						
Professional	13,968 (77.0%)	4161 (23.0%)	< 0.001	3590 (76.5%)	1105 (23.5%)	< 0.001
Manual	22,529 (70.5%)	9412 (29.5%)		6123 (69.7%)	2662 (30.3%)	
No work	836 (71.5%)	333 (28.5%)		240 (69.4%)	106 (30.6%)	
Years on current family plan						
< 1 year	9993 (98.1%)	194 (1.9%)	< 0.001	2591 (98.9%)	30 (1.1%)	< 0.001
1–5 years	15,636 (98.1%)	309 (1.9%)		3915 (98.4%)	64 (1.6%)	
> 5 years	11,765 (98.1%)	227 (1.9%)		3453 (98.8%)	43 (1.2%)	

Regarding the most recent husbands of the included women, almost two-thirds of them were aged 35 years or above (68.5%, *n* = 35179). Less than half of participants’ husbands had secondary-level education (45.6%, *n* = 23817), while 16.5% (*n* = 8200) and 18.4% (*n* = 9309) had college-level and primary-level education, respectively. A greater proportion of the participants’ husbands were manual workers (62.4%, *n* = 31941) or professional workers (35.2%, *n* = 18129), while 2.3% (*n* = 1169) had no work.

**Fig. 2:**
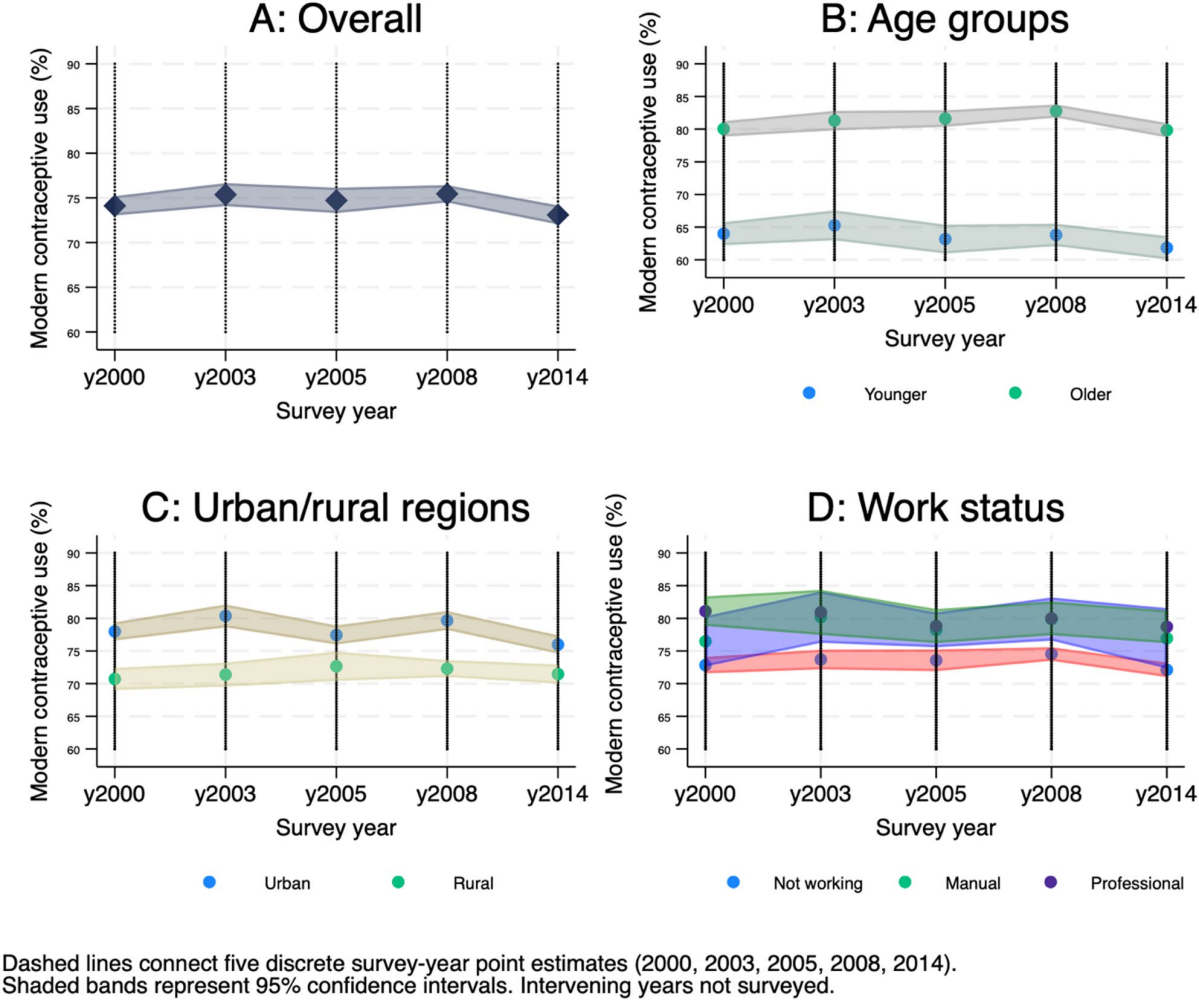
Weighted modern contraceptive use across five EDHS waves, Egypt, 2000–2014

Over survey years, the *weighted* modern contraception prevalence (MCP) was 74.4%. This varied across surveys, as illustrated in Fig. 2, being 74.1%, 75.4%, 74.7%, and 75.5% in the years 2000, 2003, 2005, and 2008, respectively. While the most recent EDHS 2014 survey showed a decline to 73.1%, down from 75.4% in 2008.

The bivariate associations between participants’ demographic, husband, and family planning factors and MCC, presenting both the pooled 5-survey data and the 2014 DHS cohort, are shown in Table 1. presenting both the pooled 5-survey data and the 2014 DHS cohort. Focusing on the 2014 survey, 9,959 out of 13,835 women (72.0%) reported current use of modern contraception. MCC use was significantly associated with women’s age group, residence, job, and wealth category (all *p* < 0.001). Notably, MCC prevalence was higher among urban residents (75.2%) compared to rural residents (69.2%) and increased alongside household wealth, reaching 75.4% among those in the “rich” (69.2%) and husbands’ age group; education level (*p* = 0.004) and work categories (*p* < 0.001) were also significantly associated with current MCC use.

Key reproductive and maternal health factors significantly associated with MCC use included the woman’s age at first marriage, the current number of living children, and the time interval between the survey and her last birth. Furthermore, preventive services, such as receiving a premarital medical exam, prenatal care, and a professional postnatal health check, were all significantly associated with higher MCC use.

Conversely, women’s own educational achievement showed no significant association with MCC use in the 2014 cohort (*p* = 0.85). Neither the mode of delivery (*p* = 0.23) nor the place of last birth (*p* = 0.068) were significantly associated with MCC use in 2014.

When analyzing the pooled data across all five DHS surveys from 2000 to 2014 (*n* = 51,329), the overall utilization of modern contraception was robust, with 37,394 (72.9%) women reported current use. Consistent with the 2014 findings, MCC use across the 14-year period was strongly associated with demographic staples such as urban residency (76.8% vs. 69.7% rural), higher household wealth, and both the woman’s and husband’s employment status (all *p* < 0.001). However, the cumulative data revealed significant associations that faded in the 2014 cohort. Notably, in the pooled dataset, a woman’s own educational achievement was significantly associated with MCC use (*p* < 0.001), as were maternal health factors like the place of delivery (*p* < 0.001) and having a Caesarean section at last birth (*p* = 0.002).

To identify factors independently associated with modern contraceptive use, a multivariable logistic regression model was fitted using data exclusively from the 2014 DHS survey (Table 2). In the unadjusted model, older participants (35-44y and 45-59y age groups), having older husbands’ age groups (35-44y and 45-59y), rich household wealth index, professional working women, ha delivering delivered in a governmental sector, and receiving their family planning methods from the private sector had higher odds of modern contraception use compared to their counterparts.

**Table 2:** Unadjusted and adjusted determinants for modern contraceptive use (2014 cohort)

	Unadjusted		Adjusted	
	OR (95% CI%) *	*P* value^	OR (95% CI%)	*P* value
Age group of respondents				
25–34	Baseline			
15–24	0.44 (0.39–0.50)	< 0.001	0.73 (0.60–0.88)	0.001
35–44	1.91 (1.71–2.13)	< 0.001	1.42 (1.18–1.70)	< 0.001
45–59	1.48 (1.24–1.76)	< 0.001	0.94 (0.71–1.25)	0.680
Husband age group				
25–34	Baseline			
14–24	0.48 (0.37–0.63)	< 0.001	1.21 (0.77–1.80)	0.392
35–44	2.01 (1.80–2.24)	< 0.001	1.03 (0.93–1.28)	0.712
45–95	2.39 (2.13–2.69)	< 0.001	0.90 (0.75–1.21)	0.397
Residence				
Rural	Baseline			
Urban	0.79 (0.71–0.88)	< 0.001	1.23 (1.01–1.49)	0.043
Household wealth index				
Poor	Baseline			
Middle	1.09 (0.95–1.24)	0.212	1.33 (1.15–1.55)	< 0.001
Rich	1.28 (1.14–1.45)	< 0.001	1.88 (1.53–2.31)	< 0.001
Respondent’s educational achievement				
No education	Baseline			
Primary	0.96 (0.81–1.15)	0.686	0.98 (0.80–1.21)	0.878
Secondary	0.88 (0.78–0.99)	0.044	0.99 (0.85–1.17)	0.970
Higher	0.84 (0.70–0.99)	0.044	0.81 (0.62–1.05)	0.110
Husband’s educational achievement				
No education	Baseline			
Primary	0.99 (0.85–1.17)	0.942	0.90 (0.74–1.10)	0.313
Secondary	0.81 (0.70–0.92)	0.002	0.79 (0.66–0.94)	0.009
Higher	0.86 (0.73–1.02)	0.084	0.75 (0.58–0.96)	0.021
Woman’s current work				
Not working	Baseline			
Manual	1.29 (0.99–1.67)	0.052	1.12 (0.85–1.47)	0.411
Professional	1.43 (1.22–1.66)	< 0.001	1.25 (1.04–1.51)	0.018
Husband’s current work				
Not working	Baseline			
Manual	0.85 (0.64–1.15)	0.298	0.78 (0.55–1.12)	0.163
Professional	1.16 (0.86–1.56)	0.333	0.98 (0.69–1.43)	0.918
The respondent had premarital examination				
No	Baseline			
Yes	0.35 (0.31–0.39)	< 0.001	0.69 (0.58–0.82)	< 0.001
Age at first marriage				
20–29	Baseline			
8–14	1.16 (0.95–1.41)	0.137	0.81 (0.64–1.02)	0.075
15–19	1.12 (1.01–1.24)	0.032	1.12 (1.04–1.35)	0.010
≥ 30	0.33 (0.24–0.45)	< 0.001	0.37 (0.24–0.55)	< 0.001
Number of living children				
1–3	Baseline			
None	0.001 (0.00-0.003)	< 0.001	0.02 (0.00-0.14)	< 0.001
> 3	1.55 (1.38–1.74)	< 0.001	1.23 (1.07–1.42)	0.004
Last birth to interview duration in years				
< 1 year	Baseline			
1–3 years	0.84 (0.73–0.97)	0.017	0.67 (0.58–0.81)	< 0.001
> 3 years	1.37 (1.19–1.56)	< 0.001	0.51 (0.43–0.64)	< 0.001
Received antenatal care (last birth)				
No	Baseline			
Yes	1.07 (0.86–1.32)	0.554	1.00 (0.80–1.27)	0.968
Delivery by caesarean section (last birth)				
No	Baseline			
Yes	0.85 (0.74–0.96)	0.012	1.23 (1.07–1.42)	0.005
Place of delivery (last birth)				
Home	Baseline			
Governmental sector	1.24 (1.01–1.54)	0.045	1.31 (1.03–1.68)	0.031
Private Sector	0.95 (0.78–1.18)	0.685	0.88 (0.68–1.13)	0.308
Others	1.20 (0.45–3.23)	0.716	1.19 (0.45–3.19)	0.726
Source for family planning				
Government	Baseline			
Private	2.36 (2.11–2.64)	< 0.001	2.49 (2.19–2.83)	< 0.001
Others	0.27 (0.16–0.46)	< 0.001	0.19 (0.10–0.33)	< 0.001

* OR: odds ratio, 95% CI: 95% confidence interval^ *P* value is significant if < 0.05

After adjusting for confounding variables, sociodemographic factors remained strongly associated with use, the odds of modern contraception use were higher among women aged 35-44y [OR (95% CI) 1.42 (1.18–1.70)] and lower among women aged 15-24y [OR (95% CI) 0.73 (0.60–0.88)] compared to women aged 25- (1.23 [1.01–1.49]) 1.23 (1.01–1.49), compared to rural-residing women. Compared to those with a poor household wealth index, the odds of modern contraception use were higher among participants with a middle household wealth index [OR (95% CI) 1.33 (1.15–1.55)] and (1.15–1.55)], and a rich household wealth index [OR (95% CI) 1.88 (1.53–2.31)]. Compared to women not working, the odds of modern contraception use were higher among participants who were professional workers [OR (95% CI) 1.25 (1.04–1.51)]. While the husband’s current work was not a significant significant predictor of women’s MCC use, his educational level was a significant one, where husbands with secondary [OR (95% CI) 0.79 (0.66–0.94)] and higher education [OR (95% CI) 0.75 (0.58–0.96)] had lower odds of their women’s MCC use compared to uneducated husbands. The odds of modern contraception use were lower for women who had a premarital examination [OR (95% CI) 0.69 (0.58–0.82)]. Additionally, the odds of MCC use were higher in women aged at 1st marriage [OR (95% CI) 15-19y 1.12 (1.04–1.35)] and lower in women aged at 1st marriage ≥ 30y [OR (95% CI) 0.37 (0.24–0.55)] compared to those who were 20–29 y at 1st marriage. Also, women with > 3 living children had higher odds of modern contraception use [OR (95% CI) 1.23 (1.07–1.42)].

Women delivered by cesarean section in the last birth were less likely to use modern contraception, 1.23 (1.07–1.42), compared to women delivered vaginally. Place of delivery was another valuable predictor, as women who delivered in the governmental sector had higher odds of modern contraception use, 1.31 (1.03–1.68), compared to those delivered at home. Women who use the private sector as their source of family planning methods had higher odds of modern contraception use, 2.49 (2.19–2.83), compared to those who depend on a governmental source.

## Discussion

This study examined trends in modern contraceptive prevalence using five nationally representative Egyptian surveys from 2000 to 2014 and identified sociodemographic and service-access factors associated with modern contraceptive use. The average MCP over the five survey waves was 74.4%, with a small range of 2.3% points. It went down from 75.4% in 2008 to 73.1% in 2014. Among the key factors found, younger women (15–24 years old), women living in rural areas, and women from low-income households were much less likely to use modern contraceptives. On the other hand, women who got birth control from the private sector were much more likely to use it (OR 2.49, 95% CI 2.19–2.83). This was also true for women who gave birth in government sector facilities, professional working women, and women who had more than three living children. In most developing countries, the use of modern contraceptives (MCP) has gone up over the past few decades [25]. MCP stayed high in Egypt over the five years of the survey (74.1%, 75.3%, 74.7%, 75.4%, and 73.1% from 2000 to 2014), showing that it stayed high within a small range of about 2.3% points.

Egypt’s mean modern contraception prevalence across the five survey waves (74.4%) considerably exceeds the WHO Eastern Mediterranean Region average of 48% and is close to the Family Planning 2030 target of 75% [26]. This difference is attributable, in part, to the denominator used: this study included only currently married, reproductive-aged women, whereas the denominator used in many comparative studies did not [27–29]. This prevalence substantially exceeds the WHO Eastern Mediterranean Region average of 48% and approaches the Family Planning 2030 target of 75%, reflecting decades of sustained governmental and non-governmental investment in family planning in Egypt [26]. The global prevalence of modern contraceptive use went up from about 48% to 49% between 2000 and 2020 [25]. Pakistan has a prevalence of 21.7%, Afghanistan 17.4%, and Sudan 5.7% [26]. Data from the region show that North African countries had an average modern contraceptive prevalence of 41.5% from 2005 to 2015 [30]. Tunisia had about 60–64%, Morocco had 42–72%, and Algeria had 49% [30, 31]. It should be noted, however, that this study’s prevalence estimates reflect use among currently married, non-pregnant, non-infecund women, all reproductive-aged women as the denominator, a broader group that mechanically lowers the prevalence estimate [27–29]. Egypt’s estimates and regional averages.

The decrease from 75.4% (2008) to 73.1% (2014) necessitates policy consideration; however, it resides within a limited range and lacks a statistically validated trend absent formal testing across all five waves. The timing coincides with the post-2011 Revolution transition period, which may have contributed to programmatic disruption; however, it cannot be excluded that the apparent decline also partly reflects the methodological differences in the 2014 EDHS sampling design (four-stage vs. three-stage approach, Sinai exclusion) rather than a true population-level reduction in modern contraceptive use. If this 2.3%-point change represents a true population-level shift rather than a methodological artifact, it would correspond to approximately 270,000 fewer users nationally, an observation that warrants further investigation through more recent data. Egypt’s overall performance is still better than that of other countries in the region, and continued investment is needed to protect these gains.

In our study, the odds of modern contraception use were higher among women aged 35–44 years old, OR (95% CI) 1.42 (1.18–1.70), and lower among women aged 15–24 years, 0.73 (0.60–0.88), compared to those aged 25–34 years old. An Ethiopian study found that women aged 25–44 were more likely to use MCC than women aged 15–19 (OR = 1.4, 1.3–1.5, P-value 0.05) [32]. Similar results were found in a study conducted in Ghana [33]. A study of sexually active girls and young women (ages 15 to 24) in Tanzania found that the use of modern contraceptives rose from 24.6% in 2004 to 32.1% in 2022 [34]. In our study, nearly half of married women in the age group of 15–24 use MCC, with a lower likelihood of usage compared to the higher age group. This observation shed light on youth, a chronically underserved cohort, and their health needs. These age-specific patterns of MCC use highlight the need for tailored family planning strategies that address the distinct reproductive intentions, behaviors, and barriers faced by younger and older women.

Furthermore, our study sheds light on childhood and teenage marriage, where 3965 (7.7%) were married at the ages of 8–14 years and 23,202 (45.2%) were married at 15–19 years of age. This finding has important implications for modern contraceptive use. Women married at younger ages often enter marriage with limited reproductive health knowledge, reduced autonomy in decision-making, and strong social expectations for early childbearing, all of which may hinder timely initiation and sustained use of modern contraceptive methods.

In our study, women residing in urban areas demonstrated a higher likelihood of utilizing modern contraception, with OR (95% CI) 1.23 (1.01–1.49) compared to their rural counterparts. The observed difference between urban and rural locations suggests that women do not have equal access to and use of modern family planning services, a distinction that warrants further investigation to disentangle supply-side availability from demand-side factors. This is because urban areas usually have more health facilities, shorter travel times, more contraceptive alternatives, and more private sector options. Women in urban areas tended to be more exposed to health information and to have greater geographic proximity to services. Whether targeted rural service delivery strategies, such as community-based distribution or task-sharing with community health workers, would reduce these observed differences warrants evaluation through program data and prospective study designs, as this cross-sectional analysis cannot establish efficacy of specific interventions.

Contraceptive use is consistently lower in low- and middle-income countries and among women from lower-wealth households within those settings [35]. Women living in urban areas and wealthy households are likely to be better served by family planning services [32, 36]. In our study, compared to those with a poor household wealth index, the odds of modern contraception use were higher among participants with a middle household wealth index of 1.33 (1.15–1.55) and a rich household wealth index of 1.88 (1.53–2.31). This is evident by exploring the report of the Family Health Initiative, 2010, where modern contraceptive use was twice as high among the wealthiest women when compared to the poorest [37].

Our study reported that the odds of modern contraception use were higher among participants who are professional workers, 1.25 (1.04–1.51), compared to non-working participants. This was consistent with findings in the DHS of Ethiopia, Rwanda, and Nepal, where working women had increased modern contraceptive use in relation to those who were not working [38]. This association may reflect greater financial independence, autonomy, and reproductive planning capacity among working women, all of which have been associated with higher use of modern contraceptive methods in the literature.

The low utilization of contraceptives among the poor women is attributed, among other factors, to costs associated with the services and related aspects [39]. In the Egyptian context, private-sector providers, including private clinics and pharmacies, typically charge market prices for contraceptive methods, while the public sector provides them free or at subsidized cost through MOHP facilities. Women accessing private-sector sources therefore tend to have higher purchasing power, explaining the strong positive association observed (adjusted OR 2.49, 95% CI 2.19–2.83). This pattern has been documented in other LMIC dual-sector health systems [10, 37],a hypothesis warranting further investigation.

Family planning counseling during premarital examination care can be an opportunity to educate women about the use of modern contraceptive methods and spacing during the childbearing period. Our study found that women who attended a premarital examination were less likely to use modern contraception. Marital examinations services are an entry point. For reproductive health education and family planning, counseling should be more systematically integrated into premarital care. Strengthening the content and quality of counseling during premarital examinations may help address misconceptions and improve subsequent contraceptive uptake among women.

Women with more than 3 living children had higher odds of MC use 1.23 (1.07–1.42) in our study, and this was supported by the study, which found that increased odds of modern contraceptive use were reported among women with a higher number of children alive compared to those with no children across the study period [39]. Particularly, the odds are highest - across the study period - among women with at least five children, followed by those with 3–4 children, and lowest among women with at most two children.

Furthermore, the women who delivered in government sector facilities showed higher odds of utilising modern contraception 1.31 (1.03–1.68) compared to home deliveries. This emphasizes the positive impact of healthcare support and counseling on family planning decisions, underscores the need to integrate family planning services into maternal and child health settings.

### Study strengths

There are several important strengths in the current study. This study utilised five successive waves of nationally representative DHS spanning two decades (2000–2014), providing strong information on long-term contraceptive patterns with significant sample sizes. The DHS approach employs systematic sampling strategies, standardised questionnaires, and quality assurance protocols to ensure high-quality, comparable data throughout survey iterations. We used the right statistical procedures that took into account the complicated survey design. Our comprehensive evaluation included a range of demographic, socioeconomic, and healthcare access characteristics identified in the literature as contributing factors influencing contraceptive use. The focus on modern contraceptive methods provides policy-relevant insights about effective family planning practices. Our emphasis on married women aligns with the sociocultural background of Egypt, where sexual behaviour is predominantly confined to marriage, thereby appropriately reflecting the population at risk of pregnancy.

### Study limitations

Several limitations should be considered. The most important thing is that the most recent data comes from EDHS 2014. At the time of the analysis, these were the most recent publicly available DHS microdata for Egypt. However, Egypt has since made big changes to its population and programmes. historical context and may not accurately depict contemporary trends. Since 2014, there have been no more EDHS studies. Future studies should focus on gathering updated nationally representative data on contraceptive use to see if the patterns found here still exist.

Second, the cross-sectional design prevents causal inference; while associations are recognised, temporal sequence cannot be determined. Third, restricting the analytical sample to non-pregnant, non-infecund married women, the population presumed to be at risk of unintended pregnancy, provides a more precise and policy-relevant prevalence estimate than studies using all reproductive-aged women as the denominator. While this approach yields higher prevalence estimates than broader denominators, it more accurately reflects true unmet need in the at-risk population, and self-reported contraceptive use is influenced by recall bias and social desirability bias.

Furthermore, the DHS contraception module captures current use based on the month preceding the survey interview, coitally-dependent methods used intermittently, such as male condoms and diaphragms, for which non-use in a given month does not necessarily reflect long-term non-adoption. Accordingly, this study’s estimates most reliably reflect use of regularly administered or long-acting methods (pills, IUDs, injectables, and implants) and may modestly undercount users of barrier methods. Also, the DHS instrument relies on women’s self-reporting of their current contraceptive method, so LAM users may not entirely meet the WHO criteria for LAM definition, representing a misclassification.

Survey intervals that last for several years make it harder to see changes that happen over a short period of time. The observed variation across surveys (74.1% to 75.4%, declining to 73.1%) spans approximately 2.3% points and should be interpreted as fluctuation within a high-prevalence plateau rather than a confirmed directional trend; formal trend testing would be required to establish statistical directionality. Furthermore, since the 2014 EDHS employed a different four-stage sampling design and excluded North and South Sinai compared to earlier waves, the apparent decline to 73.1% in 2014 may partly reflect sampling methodology differences rather than a true population-level reduction. These two explanations, political transition disruption and methodological frame change, cannot be distinguished from the available data. DHS data do not encompass counselling quality, partner attitudes, or reasons for method discontinuation, which may obscure observed associations.

Lastly, the determinant analysis only looks at data from 2014. Despite these limitations, this study presents the inaugural published cross-survey comparison of five EDHS waves, establishing a methodological benchmark for future longitudinal research.

Another limitation is that the analysis did not include fertility intentions or unmet family planning needs. The binary outcome of current contraceptive use fails to differentiate between women not using contraception due to a desire to conceive and those with a legitimate unmet need who aim to avoid pregnancy but encounter access barriers—a distinction necessitating fertility intention data and pivotal to thorough evaluations of family planning needs [28]. This constrains the interpretability of non-use as a metric of access failure. Subsequent analyses ought to integrate fertility intention and unmet need variables to furnish a more comprehensive characterisation of contraceptive behaviour in Egypt.

### Implications

These findings reveal enduring sociodemographic disparities in the utilisation of modern contraceptives in Egypt, as evidenced by five nationally representative surveys. Although the data precede 2015 and cannot demonstrate causality, the identified patterns align with the broader reproductive health literature and underscore several domains that require ongoing policy focus and additional research.

The consistently lower likelihood of modern contraceptive use among women aged 15–24, rural women, and those from low-income households suggests persistent inequities in access that have been recorded in various LMIC contexts [32, 38]. These differences suggest that aggregate modern contraceptive coverage targets (e.g., national CPR thresholds used in SDG 3.7 monitoring) may obscure significant variation among sub-groups, and that disaggregated monitoring by age, residence, and wealth quintile ought to be an integral aspect of Egypt’s family planning surveillance.

The robust correlation between private sector contraceptive sources and utilisation (OR 2.49, 95% CI 2.19–2.83), coupled with diminished odds among impoverished women, aligns with the notion that financial obstacles hinder access for the most vulnerable populations. This pattern warrants additional examination to ascertain if cost is a principal factor contributing to unmet needs in lower-income households, potentially guiding equity-oriented financing choices [35].

Women who gave birth in government-run hospitals were more likely to use modern birth control than women who gave birth at home. This suggests that using the formal health system may make it easier for people to plan their families. This connection, which has also been seen in other LMIC settings [37], suggests that it might be useful to look into how current maternal health contacts are being used for family planning in Egypt and whether there are any gaps.

The decrease from 75.4% (2008) to 73.1% (2014), which happened around the time of the political transition in 2011, needs to be looked into to see if programmatic continuity was broken during this time. To create a smart policy response, we need to know what caused the decline—whether it was supply-side, workforce, or political prioritisation factors. We can’t figure this out from the data we have now.

This study does not furnish evidence regarding the efficacy of specific interventions, and its conclusions should not be construed as directly advocating for programmatic action. Instead, the patterns found serve as a starting point for making hypotheses and show where more recent data collection, qualitative research, and program evaluation are most needed. It is highly advisable to replicate this analysis utilising EDHS 2021 microdata to ascertain whether the disparities and decline identified herein have persisted, expanded, or been mitigated.

## Conclusions

This study highlights persistent disparities in modern contraceptive use in Egypt, with notable gaps by age, residence, and socioeconomic status. Addressing these disparities requires strengthening equitable, rights-based family planning services, particularly for young women and underserved populations. Advancing Egypt’s commitments to Sustainable Development Goal 3 (SDG 3) and Family Planning (FP) 2030 will depend on sustained political will, adequate financing, and integrated reproductive health strategies that ensure universal access to modern contraception.

## Data Availability

Codes used and data analyzed from the DHS surveys are available upon request.

## Abbreviations

AOR: Adjusted Odds Ratio
CEOSS: The Coptic Evangelical Organization for Social Services
CI: Confidence interval
CPR: Contraceptive Prevalence
DHS: Demographic and Health Surveys
EDHS: Egyptian Demographic and Health Surveys
EMR: Eastern Mediterranean Region
IPUM: Integrated Public Use Microdata Series
MCC: Modern Contraception
MCP: Modern Contraceptive Prevalence
n: number
OR: Odds ratio
PSU: Primary Sampling Units
PCA: principal components analysis
SD: standard deviation
STATA: Statistics and data, a statistical software for data science
SVY: the survey prefix command in STATA software
WHO: World Health Organization
